# PM2.5‐induced pulmonary inflammation via activating of the NLRP3/caspase‐1 signaling pathway

**DOI:** 10.1002/tox.23035

**Published:** 2020-09-30

**Authors:** Hui Jia, Yang Liu, Dan Guo, Wei He, Long Zhao, Shuyue Xia

**Affiliations:** ^1^ Department of Respiratory and Critical Care Medicine Central Hospital Affiliated to Shenyang Medical College Shenyang China

**Keywords:** caspase‐1, IL‐1β, NLRP3, particulate matter, pulmonary inflammation

## Abstract

Particulate matter 2.5 (PM2.5)‐induced pulmonary inflammation has become a public concern in recent years. In which, the activation of the NLRP3/caspase‐1 pathway was closely related to the inflammatory response of various diseases. However, the promotion effect of the NLRP3/caspase‐1 pathway on PM2.5‐induced pulmonary inflammation remains largely unclear. Here, our data showed that PM2.5 exposure caused lung injury in the mice by which inflammatory cell infiltration occurred in lung and alveolar structure disorder. Meanwhile, the exposure of human bronchial epithelial cells (16HBE) to PM2.5 resulted in suppressed cell viability, as well as elevated cell apoptosis. Moreover, a higher level of inflammatory cytokine and activation of the NLRP3/caspase‐1 pathway in PM2.5‐induced inflammation mice models and 16HBE cells. Mechanistically, pretreatment with MCC950, a NLRP3/caspase‐1 pathway inhibitor, prevented PM2.5‐induced lung injury, inflammatory response, and the number of inflammatory cells in BALFs, as well as promoted cell viability and decreased inflammatory cytokine secretion. Collectively, our findings indicated that the NLRP3/caspase‐1 pathway serves a vital role in the pathological changes of pulmonary inflammation caused by PM2.5 exposure. MCC950 was expected to be the therapeutic target of PM2.5 inhalation mediated inflammatory diseases.

## 1 INTRODUCTION

Atmospheric fine particles, namely particulate matter 2.5 (PM2.5), refers to the suspended particles in the atmosphere in the aerodynamic diameter less than or equal to 2.5 μm, with small particle size, large surface area characteristics, propagation distance, the stagnation time is extended, easy access to the alveolar terminal, and easily dissolved in the blood and respiratory system.^1^, ^2^ Epidemiological studies found that the main components of PM2.5 are complex and have a carrier role, which can be loaded with sulfate, nitrate, ammonium salt, carbon‐containing particles, heavy metals, minerals, bacteria, and viruses.^3^ When PM2.5 inhaled in the lungs, it can cause an acute pulmonary inflammatory response and release a variety of inflammatory factors. At the same time, PM2.5 also contains organic compounds such as polycyclic aromatic hydrocarbons (PAHs) and lipopolysaccharides, which can produce free radicals in the lungs, break the fast oxidation and antioxidant balance, and then cause lung function damage, resulting in adverse effects of the lung.^3^ Although there are many theories on the pathogenesis of PM2.5, the mechanism and regularity of its specific harmful effects on pulmonary still need to be further explored.

Previous studies have confirmed that inflammasomes promote inflammation, and chronic inflammatory response triggered by a variety of immune cells is crucial for PM2.5‐induced pulmonary inflammation.^4^, ^5^ NOD‐like receptor protein 3 (NLRP3) inflammasome is an intracellular multi‐protein complex containing NLRP3, apoptotic speck protein (ASC), and pro‐caspase‐1.^6^ When stimulated, the NLRP3 inflammasome promotes caspase‐1 activation and subsequently converting pro‐interleukin‐1β (IL‐1β) and pro‐IL‐18 into their mature bioactive forms. Mature IL‐1β and IL‐18 are then released to extracellular space.^7^ Interestingly, as an effector of the NLRP3 inflammasome, IL‐1β is a pluripotent pro‐inflammatory cytokine involved in the process of PM2.5‐induced respiratory diseases and considered to play a pivotal role in pulmonary inflammation pathogenesis.^8^ For example, Xu et al showed that PM2.5 exposure induced IL‐1β signaling activation and resulted in pulmonary inflammation.^9^ Besides, the activation of NLRP3 inflammasome accelerated pulmonary fibrosis caused by airborne fine particulate matter.^10^ Another study has demonstrated that PM2.5 exposure resulted in neuronal injury via promoting NLRP3 inflammasome activation in a model of Alzheimer disease.^11^ The above research findings indicated that the NLRP3 inflammasome might serve an essential role in the process of pulmonary inflammation induced by PM2.5 exposure, but its functional role is not clear.

This study aimed to examine NLRP3 inflammasome was activated in pulmonary inflammation caused by PM2.5 exposure and verify whether activation NLRP3 inflammation was involved in the harmful effect on PM2.5‐induced lung injury in vivo. To do this, we used H&E to evaluate the morphological changes of the lung in the PM2.5‐induced mice models. Moreover, we further to explore the possible mechanism of PM2.5‐induced lung injury via detecting the levels of inflammatory cytokines in serum and lung tissues, and counting the number of inflammatory cells in bronchoalveolar lavage fluids (BALFs). Finally, we elucidated the effect of NLRP3 inflammasome activation on PM2.5‐induced mice pulmonary inflammation, which provided an animal experimental theoretical basis for the clinical study of lung injury caused by air pollution (ie, PM2.5).

## 2 MATERIALS AND METHODS

### 2.1 Sample collection of PM2.5

The preparation and purification methods of PM2.5 were modified according to the previous study.^12^ In brief, PM2.5 was collected in Shenyang Environmental Monitoring Centre (Shenyang, China) from December 10, 2017 and between December 17, 2018. After the collection, the glass fiber filter paper (1.5 cm × 1.5 cm) was applied to elute PM2.5 using ultrasonic vibration equipment (20 minutes × 5 times). And then, the detached PM2.5 suspension was put into a freeze‐drying bottle and placed in an ultra‐low temperature refrigerator at −80°C. Finally, the PM2.5 particles were frozen, vacuumed, and dried to dry powder and the dried PM2.5 powder was stored for standby at −20°C.

### 2.2 Animals and treatment

All experimental protocols involving animals were conducted following the guidelines of the Animal Protection Law of the People's Republic of China, 2009, and this study was performed under the approval of the Committee of Animal Research of the Central Hospital Affiliated to Shenyang Medical College. A total of 60 male BALB/c nude mice (age: 4‐5 weeks old; weight: 20 ± 2 g) were randomly separated into four groups: control group (15 mice per group), saline group (15 mice per group); PM2.5 exposure group (15 mice per group), and PM2.5 + MCC950 (the NLRP3/caspase‐1 pathway inhibitor; GLPBIO, USA; 15 mice per group) group. Mice in the PM2.5 exposure group were exposed to 120 μg/mL PM2.5 by intratracheal instillation for 14 days. Mice in the PM2.5 + MCC950 group was received 2 mg/kg MCC950 for 12 hours via tail injection before PM2.5 treatment. Mice in the control or saline group were received the same volume of air or saline via tail injection. All mice were sacrificed 15 days after the above treatment, and serum and lung tissues were collected for further experiments. Hematoxylin‐Eosin (H&E; Wanleibio, China) and immunohistochemistry (IHC; Wanleibio, China) staining were performed to observe the morphological changes of lung tissues according to the previous studies.^13^

### 2.3 Analysis of inflammatory cells in bronchoalveolar lavage fluid (BALF)

The preparation of BALF and inflammatory cell counting were analyzed according to the previous study.^14^ Briefly, the BALF of each group were collected and centrifuged with 3000 rpm for 10 minutes at 4°C. And then, 200 BALF cells were stained with Wright‐Giemsa (Wanleibio, China) and the number of inflammatory cells in BALF on each side by counting with a light microscope.

### 2.4 Cell culture and treatment

Human bronchial epithelial cells (16HBE cells) were from the Chines Academy of Sciences (Shanghai, China). 16HBE cells were cultured in bronchial epithelial cell medium (ScienCell Research Laboratories, Inc.) and incubated at 37°C in a humidified atmosphere containing 5% CO_2_. And then, 16HBE cells were seeded into a 6‐well plate and serum deprivation for 12 hours in bronchial epithelial cell medium, PM2.5 was suspended and sonicated in sterile saline to a final concentration of 1 mg/mL, and 16HBE cells were treated with 20 μg/mL PM2.5 for 24 hours.

### 2.5 Cell viability

Cell viability was assessed by 3‐(4,5‐dimethylthiazol‐2‐yl)‐2,5‐diphenyltetrazolium bromide (MTT) assay. Briefly, 24 hours after seeding into 96‐well plates (5 × 10^3^ cells/well), cells were treated with PM2.5. At 48 hours after treatment, 20 μL MTT (at a concentration of 5 mg/mL; Sigma‐Aldrich) was added, and the cells were incubated for an additional 4 hours in a humidified incubator. 200 μL DMSO was added after the supernatant discarded to dissolve the formazan. OD490 nm value was measured. The viability of the non‐treated cells (control) was defined as 100%, and the viability of cells from all other groups was calculated separately from that of the control group.

### 2.6 Cell apoptosis

2 × 10^5^ cells were maintained in an incubator with saturated humidity for 24 hours, digested with trypsin, and washed with precooled PBS three times at 4°C. ×1 binding buffer was used to resuspend the cells and adjust the cell concentration to 1 × 10^6^ cells/mL. Cells were treated with 5 μL Alexa FluorR 488 annexin V and 1 μL 100 μg/mL PI solution for 15 minutes at room temperature in dark conditions. Thereafter, 400 μL Annexin V Binding buffer was added and the cells were assessed by flow cytometry as soon as possible. The samples were analyzed with a FACScan flow cytometer (BD Biosciences, California).

### 2.7 Quantitative real‐time PCR (qRT‐PCR) analysis

Tissues were collected according to the experimental group, and the RNA extraction was performed by Trizol reagent. According to the instructions, the extracted total RNA was reverse transcribed to cDNA by PrimeScript first Strand cDNA synthesis kit (Takara, Japan). After the implementation of real‐time reverse‐transcription polymerase chain reaction, the CT value of the target gene expression was obtained compared with the control group. β‐actin was used as the internal control gene. The relative quantitative analysis was carried out by the 2^‐ΔΔCT^ method. The probes were synthesized as followed: TNF‐α: 5′‐TGGCGTGTTCATCCGTTCT‐3′ (forward) and 5′‐CCACTACTTCAGCGTCTCGT‐3′ (reverse); IL‐6:5′‐AACTCCATCTGCCCTTCA‐3′ (forward) and 5′‐CTGTTGTGGGTGGTATCCTC‐3′ (reverse); IL‐1β, 5′‐TTCAAATCTCACAGCAGCAT‐3′ (forward) and 5′‐CACGGGCAAGACATAGGTAG‐3′ (reverse); β‐actin: 5′‐GGAGATTACTGCCCTGGCTCCTAGC‐3′ (forward) and 5′‐GGCCGGACTCATCGTACTCCTGCTT‐3′ (reverse).

### 2.8 Western blot analysis

The total proteins of tissues were extracted using RIPA buffer added with Protease Inhibitor Cocktail (Cwbio, Beijing, China), incubated on ice for 30 minutes, followed by centrifuged for 15 minutes at 14000 *g*. The proteins were separated by sodium dodecyl sulfate‐polyacrylamide gel electrophoresis and then transferred to polyvinylidene difluoride (PVDF) membranes (Millipore). After being blocked with 5% defatted milk for 1 hour, the PVDF membranes were incubated with primary antibodies tumor necrosis factor‐alpha (TNF‐α; 1:500, Wanleibio, Shenyang, China), IL‐6 (1:500, Wanleibio, Shenyang, China), pro‐IL‐1β (1:500, Wanleibio, Shenyang, China), IL‐1β (1:500, Wanleibio, Shenyang, China), pro‐IL‐18 (1:500, Wanleibio, Shenyang, China), IL‐18 (1:500, Wanleibio, Shenyang, China), NLRP3 (1:500, Wanleibio, Shenyang, China), apoptotic speck protein (ASC; 1:500, Wanleibio, Shenyang, China), and caspase‐1 (1:500, Wanleibio, Shenyang, China) and β‐actin (1:1000, Wanleibio, Shenyang, China) overnight at 4°C. The membranes were incubated with horseradish peroxidase‐coupled secondary antibody (IgG‐HRP, 1:5000, Wanleibio, China) for 1 hour at room temperature before being revealed using enhanced chemiluminescence reagent (Engreen, Beijing, China). The ECL Western blot detection kit (Bio‐Rad) was employed to detect the optical density of the protein bands to evaluate the expression levels of the protein proteins.

### 2.9 Measurement of inflammatory cytokines, TP, and LDH by ELISA

The enzyme‐linked immunosorbent assay (ELISA) kits were used to detect the expression level of IL‐6 (Wanleibio, China), IL‐1β (Wanleibio, Shenyang, China), TNF‐α (Wanleibio, Shenyang, China), Lactic dehydrogenase (LDH; Wanleibio, Shenyang, China), and total protein (TP; Nanjing Jiancheng Bioengineering Institute, China) in mice serum according to the manufacturer instructions.

### 2.10 Statistical analysis

All experiments were repeated at least three times. Data were presented with mean ± *SD*. Statistical analysis was performed by GraphPad Prism 8 (GraphPad Software, Inc.), and differences between groups were analyzed by one‐way ANOVA followed by Tukey post hoc test. Student *t* test was used for comparisons between two groups. *P* < .05 was considered to be statistically significant.

## 3 RESULTS

### 3.1 PM2.5 exposure induced lung damage in vivo

**FIGURE 1:**
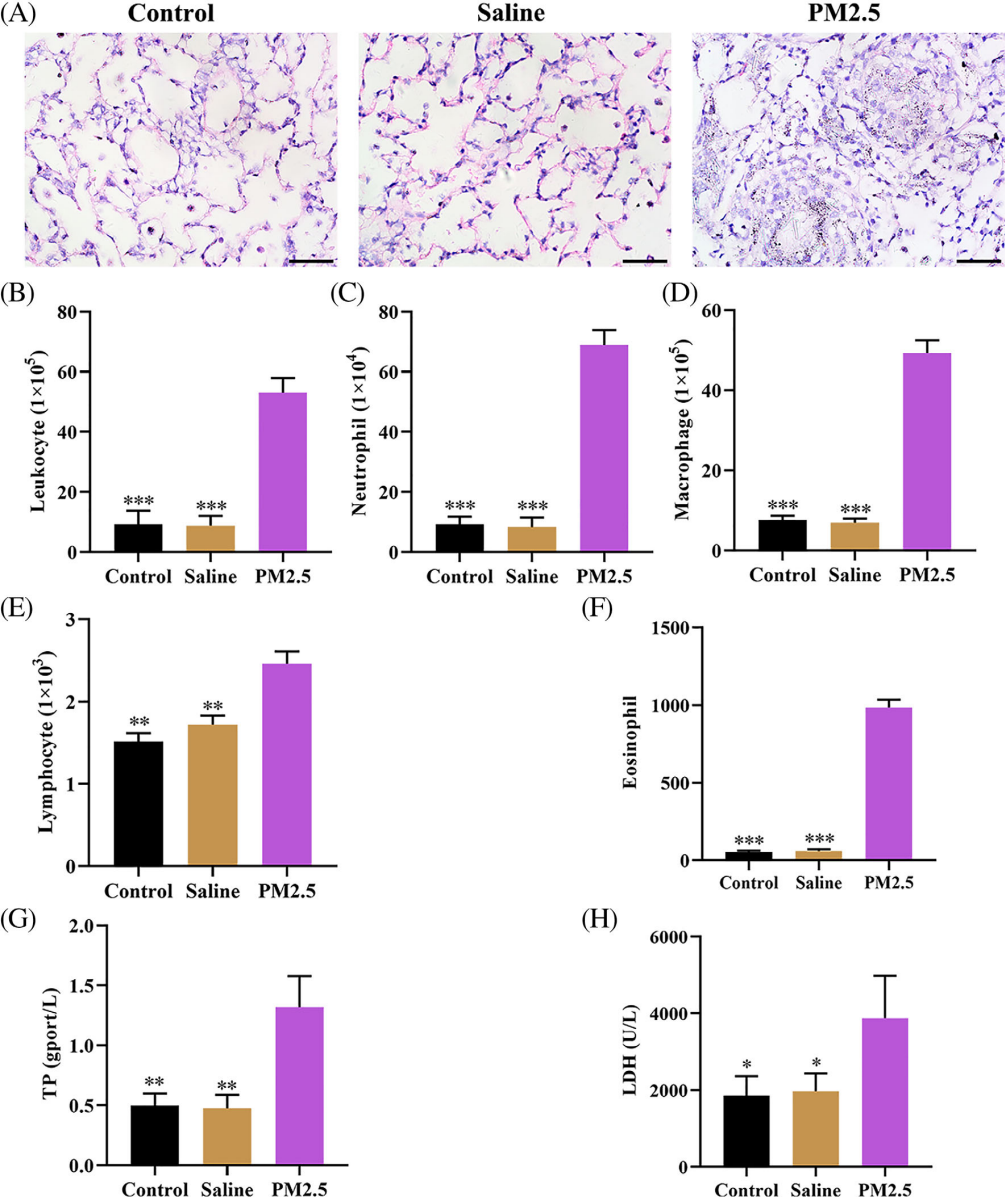
PM2.5 exposure triggered lung injury and enhanced the number of inflammatory cells in BALFs. A, All mice exposure to PM2.5 (120 μg/mL) by intratracheal instillation for 14 days, H&E staining was used to observe the histological changes of the lung (magnification, ×400); B‐F, BALF cell numbers in leukocyte, neutrophil, macrophage, lymphocyte, and eosinophil were analyzed; G and H, ELISA assay was applied to detect the levels of TP and LDH in the serum of PM2.5 exposure to mice. ^*^ *P* < .05, ^**^ *P* < .01, ^***^ *P* < .001, compared with the PM2.5 exposure group [Color figure can be viewed at wileyonlinelibrary.com]

Previous studies have confirmed that PM2.5 exposure could cause acute airway inflammation.^15^, ^16^ In the present study, we explore the effect of PM2.5 on pulmonary inflammation in vivo. H&E staining showed that compared with the control and saline group, PM2.5 treatment (120 μg/mL) resulted in alveolar structure disorder, alveolar septal thickening, erythrocyte effusion, and infiltration of inflammatory cells (Figure 1), suggesting that PM2.5 exposure triggered to lung injury and inflammatory response. Next, we examine the BALF cell numbers in leukocytes, neutrophils, macrophages, lymphocytes, and eosinophils derived from the mice treated with PM2.5. The data revealed that PM2.5 exposure enhanced the number of leukocytes, neutrophils, macrophages, lymphocytes, and eosinophils compared with the control group and saline group (Figure 1B‐F). Moreover, previous studies have confirmed that PM2.5‐induced oxidative stress appears as an excessive variation of associated‐enzyme activities, oxidant accumulation and so on.^17^, ^18^ As expected, ELISA analysis results showed that the levels of TP and LDH in the PM2.5 treatment group were higher than those in the control and saline group (Figure 1G,H). Taken together, lung injury after chronic exposure to PM2.5 was related to the imbalance inflammatory response.

### 3.2 PM2.5 exposure promoted inflammatory response via activating the NLRP3/caspase‐1 pathway

**FIGURE 2:**
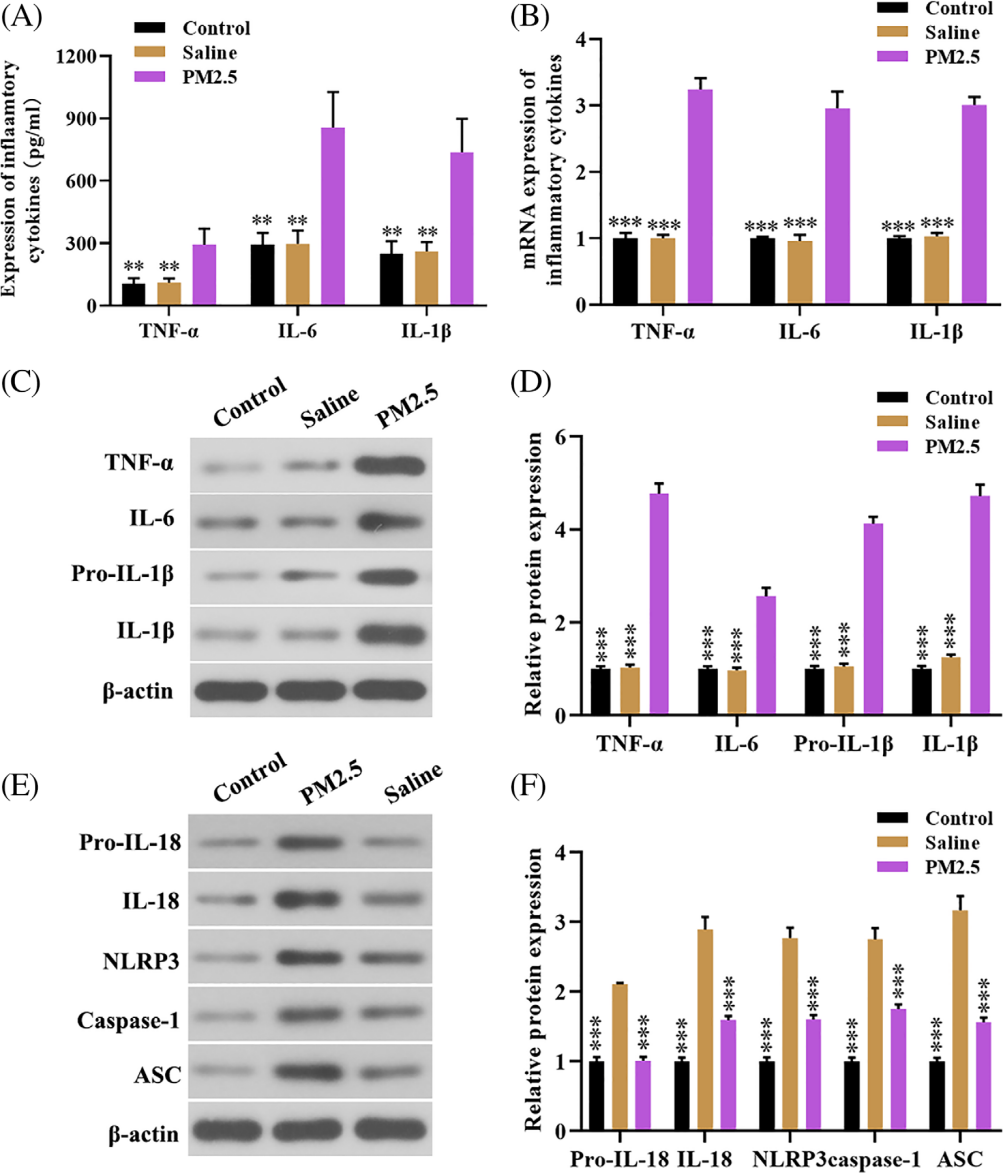
PM2.5 exposure increased the expression levels of inflammatory cytokines and activated the NLRP3/Caspase‐3 pathway. A, The levels of TNF‐α, IL‐1β, and IL‐6 in the serum of mice treated with PM2.5; B‐D, The mRNA and protein level of TNF‐α, IL‐1β, and IL‐6 in lung tissues of PM2.5 exposure to mice were determined using qRT‐PCR and western blot; E‐F, Western blot was performed to detect the protein levels of NLRP3/caspase‐1 pathway‐related genes (IL‐18, NLRP3, caspase‐1, and ASC) in lung tissues. **P < .01, ***P < .001, compared with the PM2.5 exposure group [Color figure can be viewed at wileyonlinelibrary.com]

Previous studies showed that activation of the NLRP3/caspase‐1 pathway contributed to the inflammatory response in the development of various diseases, including pulmonary fibrosis, airway inflammation, and chronic obstructive pulmonary diseases.^19^, ^20^ In the present study, we examined the effect of PM2.5 exposure on the levels of inflammatory cytokines and NLRP3 inflammasome activation related genes. As shown in Figure 2A, PM2.5 exposure increased the expression levels of serum inflammatory cytokines compared with the control and saline group. Similarly, a higher mRNA and protein levels of TNF‐α, IL‐1β, and IL‐6 in lung tissues of mice treated with PM2.5 (Figure 2B‐D). Moreover, western blot showed that PM2.5 treatment enhanced the protein levels of NLRP3, caspase‐1, IL‐1β, and IL‐18 compared with the control group and saline group (Figure 2E,F). Taken together, the NLRP3/caspase‐1 pathway was activated in PM2.5 exposure inflammation mice model.

### 3.3 Inhibition of the NLRP3/caspase‐1 pathway by MCC950 alleviated pulmonary inflammation in PM2.5‐induced mice model

**FIGURE 3:**
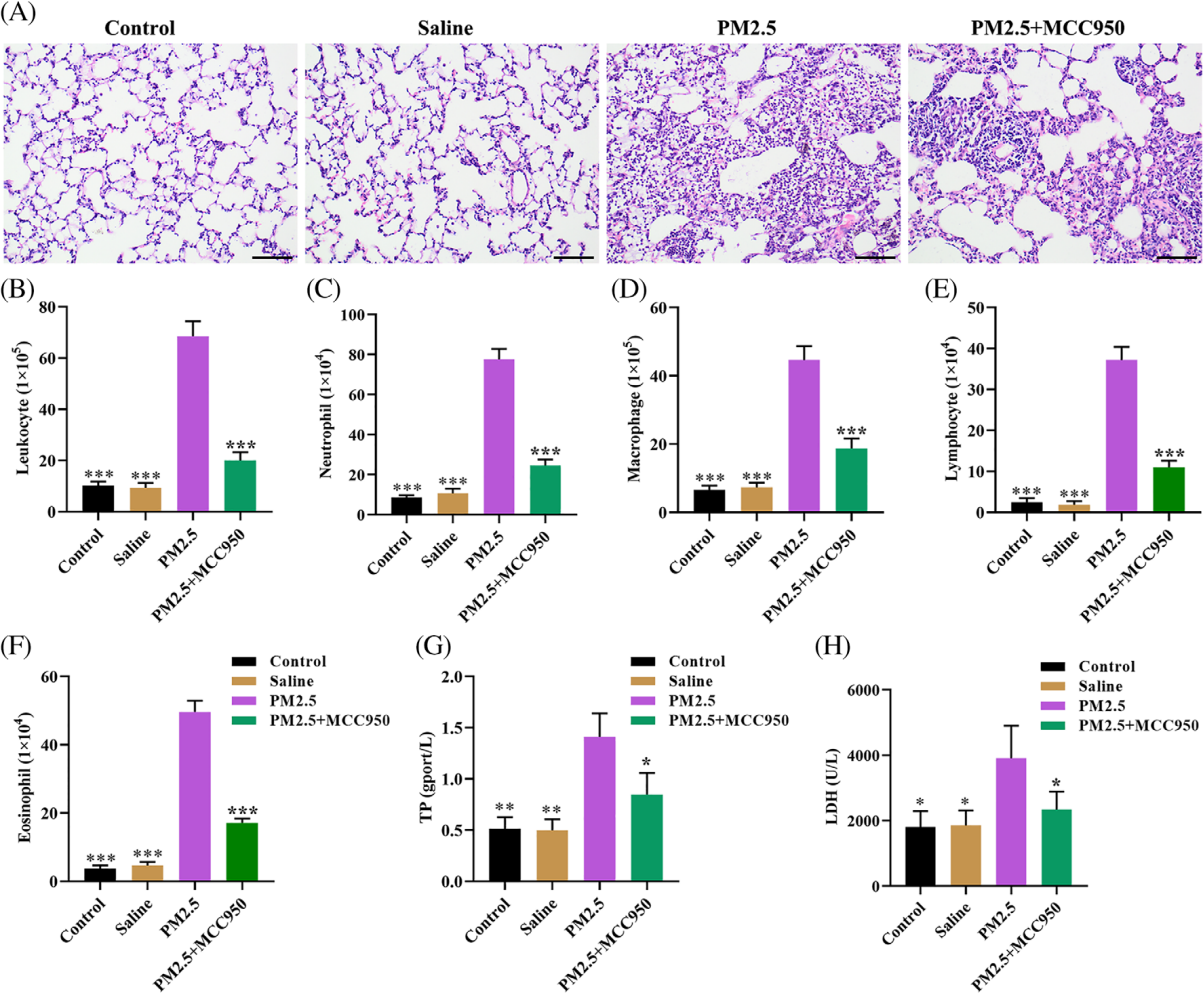
Inhibition of the NLRP3/caspase‐1 pathway alleviated PM2.5‐induced lung injury and decreased the number of inflammatory cells in BALFs. A, Mice in the PM2.5 + MCC950 group was received 2 mg/kg MCC950 for 12 hours via tail injection before PM2.5 treatment; H&E staining was used to observe the histological changes of the lung (magnification, ×200); B‐F, BALF cell numbers in leukocyte, neutrophil, macrophage, lymphocyte, and eosinophil were analyzed; G and H, ELISA assay was applied to detect the expression levels of TP and LDH in the serum of mice. *P < .05, **P < .01, ***P < .001, compared with the PM2.5 exposure group [Color figure can be viewed at wileyonlinelibrary.com]

To explore the role of the NLRP3/caspase‐1 pathway in PM2.5‐induced lung inflammation in mice model, we inactivation of the NLRP3/caspase‐1 pathway by pharmacological inhibitor MCC950, and the results showed that MCC950 treatment significantly improvement in alveolar structure disorder induced by PM2.5 in vivo (Figure 3A). Subsequently, pretreatment with MCC950 significantly decreased the number of inflammatory cell count in BALFs, including leukocytes, neutrophils, macrophages, lymphocytes, and eosinophils compared with the PM2.5 exposure alone group (Figure 3B‐F). Moreover, MCC950 treatment notably reduced PM2.5‐induced the levels of TP and LDH (Figure 3G,H). Meanwhile, there was no significant difference between the MCC950 + PM2.5 group and the control or saline group. Collectively, the inactivation of the NLRP3/caspase‐1 pathway by MCC950 alleviated lung inflammation induced by PM2.5 exposure in mice model.

### 3.4 MCC950 administration ameliorated inflammatory response in PM2.5‐induced mice model

**FIGURE 4:**
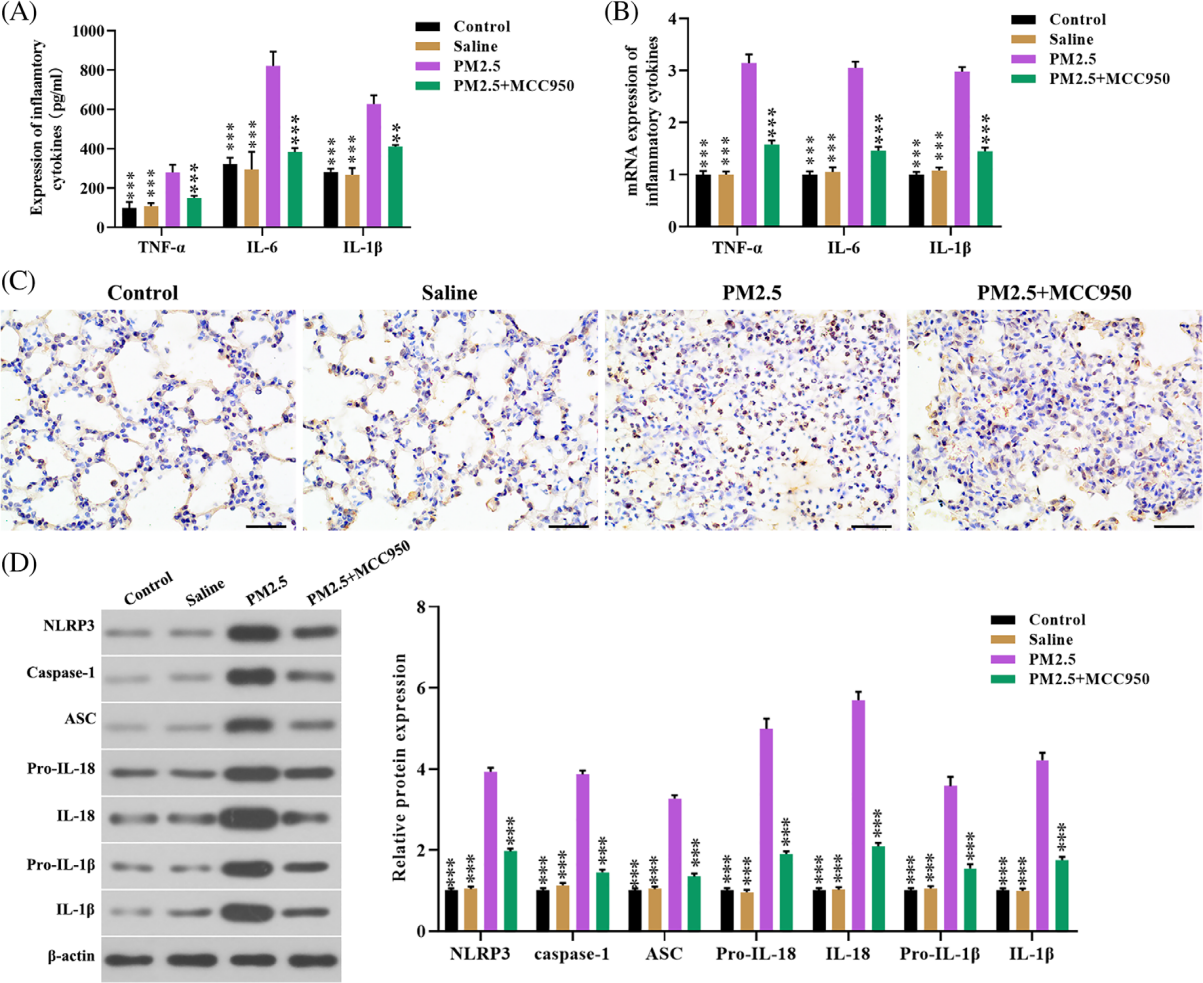
Inhibition of NLRP3/caspase‐1 pathway attenuated PM2.5‐induced pulmonary inflammation. A, The levels of TNF‐α, IL‐1β, and IL‐6 in the serum of mice treated with PM2.5 alone and pretreated MCC950 before PM2.5 exposure; B, the mRNA level of TNF‐α, IL‐1β, and IL‐6 in lung tissues were determined using qRT‐PCR; C, IHC staining was performed to examine the expression of NLRP3 in lung tissues (magnification, ×400); D, Western blot was performed to detect the protein levels of the NLRP3/caspase‐1 pathway‐related genes in lung tissues. ***P < .001, compared with the PM2.5 exposure group [Color figure can be viewed at wileyonlinelibrary.com]

To further investigate the effect of MCC950 on pulmonary inflammation induced by PM2.5 in vivo. As shown in Figure 4A,B, the expression of TNF‐α, IL‐6, and IL‐1β in serum and lung tissues of mice treated with pharmacological inhibitor MCC950 before PM2.5 exposure was significantly decreased. Moreover, IHC staining showed that MCC950 treatment significantly reduced the expression of NLRP3 in lung tissues of mice exposure to PM2.5 (Figure 4C). Furthermore, the protein levels of NLRP3, ASC, caspase‐1, and IL‐18 in lung tissues of mice treated with PM2.5 alone or MCC950 prior to PM2.5 exposure were determined by western blot. As expected, MCC950 treatment significantly decreased the protein levels of NLRP3, ASC, caspase‐1, and IL‐18 in lung tissues (Figure 4D). These data indicate that inhibition of the NLRP3/caspase‐1 pathway by MCC950 ameliorated PM2.5‐induced pulmonary inflammation in vivo.

### 3.5 PM2.5‐induced cell apoptosis in 16HBE cells

**FIGURE 5:**
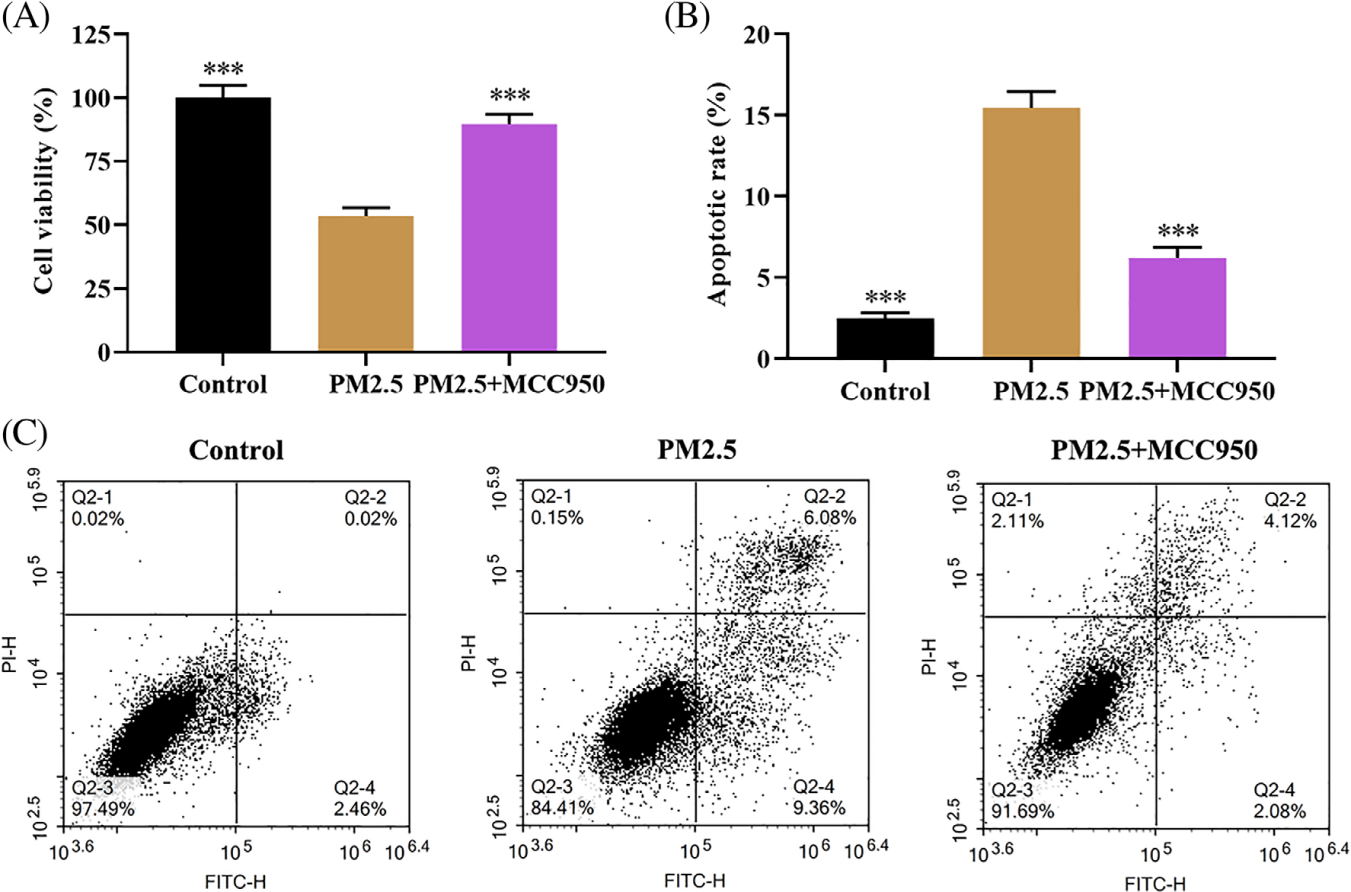
PM2.5 exposure induced cell apoptosis and reduced cell viability in 16HBE cells. A, MTT assay was employed to detect cell viability; B and C, flow cytometry was performed to examine the apoptosis of 16HBE cells. ***P < .001, compared with the PM2.5 treatment group [Color figure can be viewed at wileyonlinelibrary.com]

To further verify the effect of PM2.5 on airway epithelial cell viability in vitro, 16HBE cells were cultured with normal media and PM2.5 for 24 hours. MTT assay showed that PM2.5 treatment significantly suppressed the proliferation of 16HBE cells compared with the control group (Figure 5A). Moreover, the effect of PM2.5 on apoptosis was examined by flow cytometry. As expected, compared with the control group, the proportion of apoptotic cells of PM2.5‐treated 16HBE cells showed a significant increase (Figure 5B, C). Taken together, PM2.5 reduced cell viability and promoted apoptosis of 16HBE cells.

### 3.6 PM2.5 treatment increased the levels of inflammatory cytokine in vitro via activating the NLRP3/caspase‐1 pathway

**FIGURE 6:**
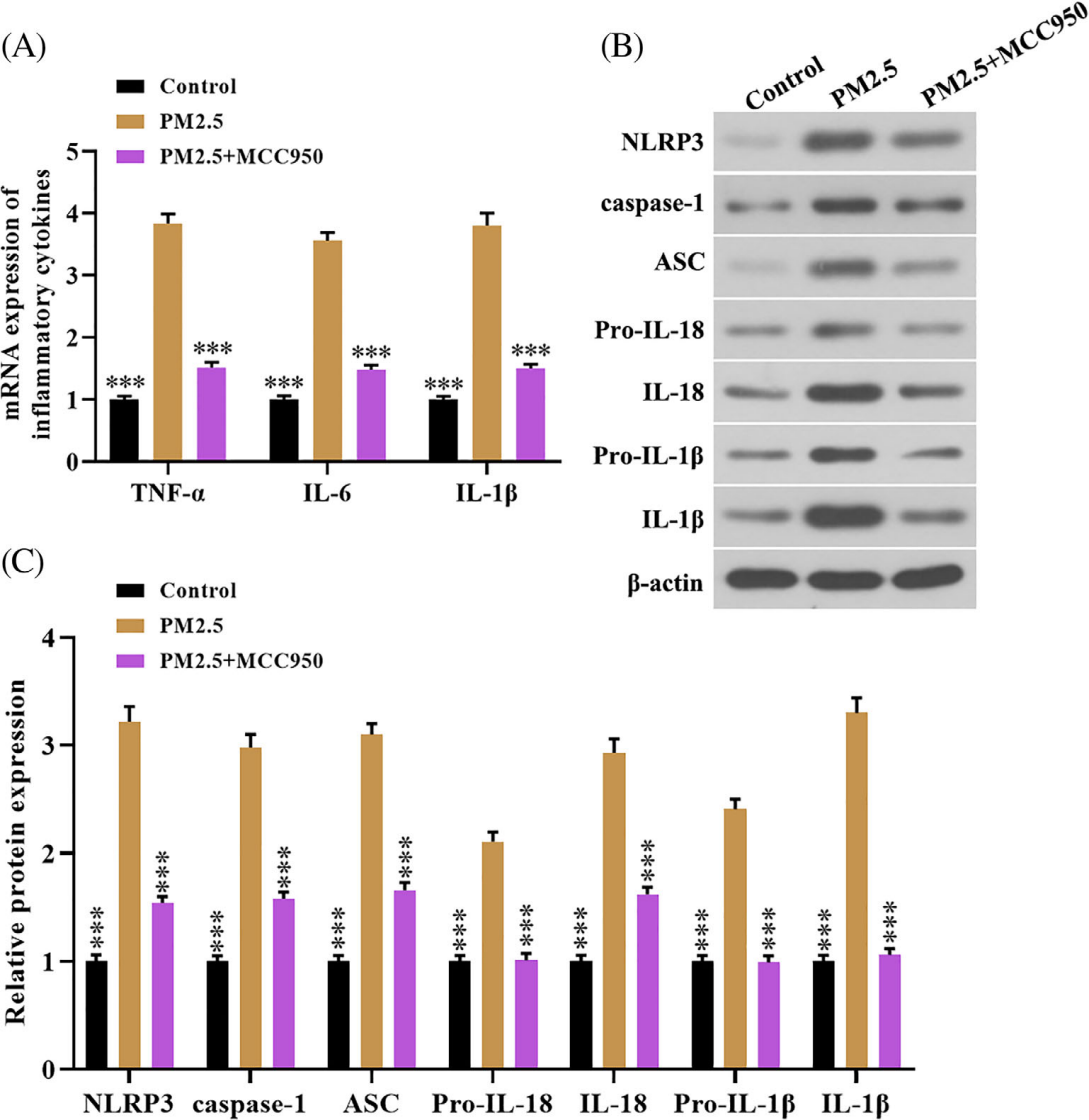
PM2.5 induced inflammation through activation of the NLRP3/caspase‐1 pathway in 16HBE cells. A, qRT‐PCR was used to detect the mRNA levels of TNF‐α, IL‐6, and IL‐1β in 16HBE cells; B and C, Western blot was employed to detect protein levels of NLRP3, caspase‐1, ASC, pro‐IL‐18, IL‐18, pro‐IL‐1β, and IL‐1β in 16HBE cells [Color figure can be viewed at wileyonlinelibrary.com]

To examine whether PM2.5 induced inflammatory response airway epithelial cells through the NLRP3/caspase‐1 pathway in vitro. As shown in Figure 6A, PM2.5 treatment significantly promoted the levels of TNF‐α, IL‐6, and IL‐1β. Moreover, western blot assay showed that PM2.5 up‐regulated the protein levels of NLRP3, ASC, caspase‐1, IL‐1β, and IL‐18 compared with the control group (Figure 6B, C). Importantly, MCC950 treatment markedly alleviated the promotion effect of PM2.5 on inflammation (Figure 6). Collectively, PM2.5 induced inflammation through activation of the NLRP3/caspase‐1 pathway in 16HBE cells.

## 4 DISCUSSION AND CONCLUSIONS

In recent years, air pollution is grim, and the haze occurrences frequently. And PM2.5 was one of the important constituents in the haze, plays the most important role in causing adverse health effects. Studies have shown that most respiratory diseases are closely related to atmospheric environmental particulate matter, and atmospheric PM2.5 has a significant effect on the development of respiratory diseases.^21^ In the present study, our data showed that PM2.5 exposure induced lung damage, and enhanced the number of inflammatory cells in BALFs and inflammatory cytokines secretion via activating the NLRP3/caspase‐1 pathway. Moreover, inhibition of the NLRP3/caspase‐1 pathway by pharmacological inhibitor MCC950 ameliorated pulmonary inflammation caused by PM2.5 exposure in vivo and in vitro.

Increasing evidence has confirmed that pulmonary inflammation caused by PM2.5 exposure was related to inflammatory cell recruitment in the alveolus and be activated.^22^ Ogino et al confirmed that PM2.5‐induced airway inflammation via increasing the BALFs cell numbers in total cell fractions, macrophages, neutrophils, eosinophils, and lymphocytes.^12^ Zheng et al found that PM2.5 exposure significantly increased the number of neutrophilic and the levels of the Th2 cytokines in BALFs.^4^ Moreover, another study has confirmed that the number of infiltrated macrophages and neutrophils in the lung of PM2.5‐treated mice was enhanced,^23^ as well as PM2.5‐induced activated RAW264.7 macrophages inflammatory factors release.^24^ In line with previous studies, our data showed that the number of inflammatory cells in BALFs were enhanced in the alveolus of mice exposure to PM2.5.

It has demonstrated that exposure to PM2.5 contributed to increasing the release of IL‐1β, an inflammatory effector, which has been used as a target for the treatment of inflammatory‐related diseases.^9^, ^10^, ^25^ In the present study, our data showed that PM2.5 exposure increased the expression of inflammatory cytokines, including IL‐1β, IL‐6, and TNF‐α in lung tissues compared with the control and saline group. Moreover, previous studies found that the expression level of IL‐1β was regulated by multiple pathways in inflammation diseases, such as toll‐like receptor 4 (TLR4)/nuclear factor kappa beta (NF‐κB) pathway,^26^, ^27^ NLRP3 inflammasome,^28^ Akt pathway.^13^ For instance, Wang et al showed that PM2.5 exposure enhanced the levels of IL‐1β by activating the TLR4/MyD88 pathway and NLRP3 inflammasome in murine airway inflammation.^29^ We found that inhibition of the NLRP3/caspase‐1 pathway by MCC950 significantly attenuated PM2.5‐induced IL‐1β release in lung tissues and serum of mice. Furthermore, NLRP3 inflammasome activation was associated with particulate matter induced pulmonary fibrosis,^10^, ^30^ and the activation of NLRP3 inflammasome can promote caspase‐1 activation and subsequently converting pro‐IL‐1β into its mature bioactive forms. Meanwhile, inactivation of NLRP3 inflammasome contributed to alleviating the progression of inflammatory diseases, including pulmonary fibrosis,^31^ murine allergic airway inflammation,^32^, ^33^ myocardial dysfunction,^34^ and cardiovascular diseases,^35^ etc. Collectively, suppression of NLRP3 inflammasome activation may be a critical therapeutic target in PM2.5‐induced pulmonary injury.

In conclusion, our data revealed that a pioneering study on the regulation of NLRP3 inflammasome on PM2.5‐induced pulmonary inflammation. Inhibition of NLRP3 inflammasome activation decreased the number of inflammatory cells in BALFs and the generation of inflammatory cytokines triggered by PM2.5 exposure in vivo. In addition, our further study should be focus on the functional role of NLRP3 inflammasome in the regulation of PM2.5‐mediated pulmonary diseases and examine whether NLRP3 inflammasome involved in the process of PM2.5‐induced chronic lung injury related diseases via regulating other functional genes. Simultaneously, we should seek an effective drug to inactivation of NLRP3 inflammasome and verify NLRP3 inflammasome function via multi‐level experiments (ie, in vitro and in vivo) to provide clinical evidence and treatment target for PM2.5 exposure induced lung diseases.

## AUTHOR CONTRIBUTION STATEMENT

Hui Jia and SYX designed this study. Yang Liu and Dan Guo collected and analyzed the data. Yang Liu, Wei He, and Long Zhao checked the statistical analysis. Hui Jia wrote the manuscript. Shuyue Xia supervised this manuscript.

## CONFLICT OF INTEREST

The authors declare no conflict of interests.
